# In silico Designed Ebola Virus T-Cell Multi-Epitope DNA Vaccine Constructions Are Immunogenic in Mice

**DOI:** 10.3390/vaccines7020034

**Published:** 2019-03-29

**Authors:** Sergei I. Bazhan, Denis V. Antonets, Larisa I. Karpenko, Svetlana F. Oreshkova, Olga N. Kaplina, Ekaterina V. Starostina, Sergei G. Dudko, Sofia A. Fedotova, Alexander A. Ilyichev

**Affiliations:** State Research Center of Virology and Biotechnology “Vector”, Koltsovo, 630559 Novosibirsk Region, Russia; antonec@nprog.ru (D.V.A.); karpenko@vector.nsc.ru (L.I.K.); sv_oresh@mail.ru (S.F.O.); okaplina@vector.nsc.ru (O.N.K.); starostina_ev@vector.nsc.ru (E.V.S.); s.g.dudko@gmail.com (S.G.D.); hz.smoke.on.the.water@gmail.com (S.A.F.); ilyichev@vector.nsc.ru (A.A.I.)

**Keywords:** ebola virus disease, artificial T-cell antigens, DNA vaccine constructs, computer design, gene expression, immunogenicity

## Abstract

*Background*: The lack of effective vaccines against Ebola virus initiates a search for new approaches to overcoming this problem. The aim of the study was to design artificial polyepitope T-cell immunogens—candidate DNA vaccines against Ebola virus and to evaluate their capacity to induce a specific immune response in a laboratory animal model. *Method*: Design of two artificial polyepitope T-cell immunogens, one of which (EV.CTL) includes cytotoxic and the other (EV.Th)—T-helper epitopes of Ebola virus proteins was carried out using original TEpredict/PolyCTLDesigner software. Synthesized genes were cloned in pcDNA3.1 plasmid vector. Target gene expression was estimated by synthesis of specific mRNAs and proteins in cells transfected with recombinant plasmids. Immunogenicity of obtained DNA vaccine constructs was evaluated according to their capacity to induce T-cell response in BALB/c mice using IFNγ ELISpot and ICS. *Results*: We show that recombinant plasmids pEV.CTL and pEV.Th encoding artificial antigens provide synthesis of corresponding mRNAs and proteins in transfected cells, as well as induce specific responses both to CD4+ and CD8+ T-lymphocytes in immunized animals. *Conclusions*: The obtained recombinant plasmids can be regarded as promising DNA vaccine candidates in future studies of their capacity to induce cytotoxic and protective responses against Ebola virus.

## 1. Introduction

Ebola fever or Ebola virus disease (EVD) is an acute disease resulting in high rates of mortality. It is caused by RNA-containing viruses of *Filoviridae* family, genus *Ebolavirus.* Viruses of genus *Ebolavirus* belong to five species with different fatality rates and serologic properties: Zaire ebolavirus, Sudan ebolavirus, Bundibugyo ebolavirus, Tai Forrest ebolavirus, and Reston ebolavirus. The first outbreaks of EVD were registered in 1976 initially in Zaire (currently the Democratic Republic of the Congo) in the Ebola river area (Zaire species, genus Ebola) and almost concurrently in Sudan (Sudan species, genus Ebola). After that, sporadic outbreaks were registered over a period of 40 years in Central Africa countries, affecting from one to several dozens or even hundreds of people. All those outbreaks were successfully and timely controlled. The Ebola fever outbreak in Western Africa in 2014–2015 was found to be significantly extensive. To eliminate it, efforts of several countries across the world were required [1].

The main problems the doctors met with controlling Ebola fever included the absence of a vaccine and prophylactic drugs against this disease. Despite the high fatality rate, an epidemic danger of this agent was always believed to be insignificant. Expensive development of vaccines and therapeutic drugs against rare although lethal disease in each case seemed to be unprofitable and attracted interest only due to a potential bioterrorism threat. The 2014–2015 Ebola fever outbreak claimed more than 11 thousand lives, which enforced studies on countermeasures against this infection. Currently, active studies on development of control measures against the virus are being carried out including small interfering RNA, low-molecular compounds, and antibodies [2,3], drugs based on monoclonal antibodies [3], and, certainly, vaccines. There are a number of approaches to designing vaccines against Ebola virus including DNA vaccines, subunit vaccines, as well as vaccines based on virus-like particles and viral vectors such as adenoviruses HAdV-5, HAdV-26, ChAdV-3, vesicular stomatitis virus (VSV), human cytomegalovirus, and modified vaccinia virus Ankara (MVA) [4,5,6]. Their protective efficacy was evaluated in non-human primate models. Furthermore, to date, several vaccines to control the virus in humans were described, i.e., rVSV-ZEBOV [7], Ad5-ZEBOV [8], GamEvac-Combi [9], and others.

The majority of developed experimental vaccines were constructed based on genetically modified viruses encoding full-length viral antigens that induce responses of both antibodies and cytotoxic T-lymphocytes (CTL) [10]. However, it should be noted that data on the protective effect of neutralizing antibodies against filoviruses obtained in studies on NHP are contradictory. It was shown that some antibodies protect animals against further infection but fail to neutralize the virus, while others neutralize the virus but fail to protect animals [11]. Consequently, the relative significance of neutralizing antibodies compared with those that can provide protection using other mechanisms (e.g., antibody-dependent cell cytotoxicity or Fc-dependent mechanisms) is still unclear. Besides this, the question deserves to be asked about the role of non-neutralizing antibodies during protection against Ebola virus, considering the well-known effect of antibody-dependent enhancement of infection [12].

A number of Ebola virus vaccine candidates develop base on glycoprotein (GP). However, antibodies induced by such GP vaccines are typically autologous and limited to the other members of the same species. In contrast, T-cell vaccines designed on the basis of conservative regions of the filovirus proteins can protect against different members of the filovirus family. It was shown that simian adenovirus- and poxvirus MVA-vectored vaccines encoding cross-filovirus immunogen provided broad immunogenicity and a solid protection of the BALB/c and C57BL/6J mice against high, lethal challenges with Ebola and Marburg viruses, two distant members of the family [5].

Another promising trend in virus T-cell vaccine design is DNA vaccine. Compared with virus-vectored vaccines, DNA vaccines demonstrate a number of advantages [13]. They are inexpensive, non-infective, and simply produced in large quantities; they can be reused since previously existing immunity is of no importance for DNA-vectors compared to viral vectors. In addition, DNA-vaccination provides the most natural way of antigen presentation by both MHC class I and class II molecules, focusing the immune response only on antigen of interest, providing long-term persistence of immunogenicity, polarizing T-helper cells toward type 1 or type 2, and inducing protective humoral and cellular immune responses. The other important feature of DNA vaccines is the ability to put several antigens or several epitopes from different antigens in the plasmid, resulting in immunization against all of the agents; and a mixture of DNA plasmids can be used to form a broad spectrum of vaccines. At last, in vivo expression of gene of interest ensures the protein resembles the normal eukaryotic structure more closely, with accompanying post-translational modifications. The only disadvantage of DNA vaccine is their relatively low immunogenicity; thus, they require administration of several doses to achieve the desirable immunity [14]. However, this disadvantage can be evaded at present due to the strategy of intramuscular electroporation making it possible to significantly enhance DNA-vaccination efficiency [15,16,17]. In addition, to increase the immunogenicity of DNA vaccines it is possible to use other methods for delivery, for example, using VLPs [18] that include DNA plasmid, as well as by translating DNA vaccines into RNA vaccine format [19].

One of the promising trends in virus vaccine design is DNA vaccine encoding artificial polyepitope immunogens. These vaccines comprise a combination of conservative cytotoxic T-lymphocytes (CTL)- and T-helper (Th)-epitopes selected from different viral proteins and combined in one molecule [20,21,22]. Progress in identifying T-cell epitopes, as well as understanding the mechanisms of processing and presentation of antigens by Major Histocompatibility Complex (MHC) class I and II pathways are instrumental for rational designing of artificial polyepitope vaccines inducing responses of cytotoxic (CD8+) and helper (CD4+) T-lymphocytes [20,22,23]. 

This study aims to design artificial polyepitope T-cell immunogens—candidate DNA vaccines against Ebola virus using computer-aided molecular design, and to study their capacity to induce a specific immune response in a laboratory animals model.

## 2. Materials and Methods

### 2.1. Software

Selection of known T-cell epitopes of Ebola viruses was carried out based on the IEDB—Immune Epitope Database (http://iedb.org) [24]. Prediction of T-cell epitopes was conducted using TEpredict software [25]. Design of polyepitope antigens was performed with PolyCTLDesigner [26]. Genes encoding target immunogens were developed using GeneDesigner software [27]; a compound of codons was optimized to achieve high expression of genes in human cells. Analysis of amino acid sequences of peptides, evaluating their conservatism, statistical analysis of obtained findings, and graph plotting were executed in statistical analysis environment R (version 3.2; https://www.R-project.org/, Vienna, Austria) [28].

### 2.2. Gene Synthesis and Cloning

Designed genes were synthesized (CJSC Eurogen, Moscow, Russia) and then cloned into pcDNA3.1 eukaryotic plasmid vector and sequenced. The obtained recombinant plasmids pEV.CTL and pEV.Th—candidate DNA vaccines against Ebola virus were used to prove the expression of designed target genes in eukaryotic cells and to assess their immunogenicity in mice of the BALB/c line.

### 2.3. Evaluation of Target Gene Transcription

Specific mRNA synthesis of target genes was evaluated in eukaryotic cells 293T transfected with pEV.CTL and pEV.Th using MATra-A reagent according to the manufacturer’s instruction (PromoKine, Heidelberg, Germany). Cells were cultured in Dulbecco’s Modified Eagle’s Medium (DMEM) with 10% FBS. 48 h after transfection mRNAs were isolated from cells with a kit for RNA isolation (Promega, USA). Before reverse transcription all RNA samples were treated with RNase-free DNase. cDNAs were obtained by reverse transcription using RevertAid H Minus First Strand cDNA Synthesis Kit (Thermo Scientific, Germany). Further, the obtained cDNA carried out PCR with the use of specific primers to gene *EV.CTL* (f^CTL^—AACTCAGGCACTCTTCCTGC, r^CTL^—TCGTACCGGAATCTCAGGGT) and gene *EV.Th* (f^Th^—ACGTTGACAAGCTGAGGAGG, r^Th^—GAGAGTCCTCAGCCCAGAGA). The amplification product was analyzed by electrophoresis in 1% agarose gel. 

### 2.4. Immunochemical Staining of Products of Transfected Cells

The presence of target proteins in eukaryotic cells 293T transfected with pEV.CTL and pEV.Th was detected through immunostaining. Cell transfection was carried out using MATra-A reagent according to the manufacturer’s instruction (PromoKine, Germany). Cells were cultured in DMEM medium with 10% FBS. 32 h after transfection cells were washed in phosphate buffer solution (PBS), fixed in a mix of ice methanol/acetone (1:1) at 40 °C for 30 min, and then washed in PBS again. The expression products of *EV.CTL* and *EV.Th* gene were detected in immunochemical staining. Staining was carried out using antibodies MAT 29F2/30A6 (JSC Vector-Best, Novosibirsk, Russia) to marker epitope EPFRDYVDRFYKTL being a part of all constructs, and using conjugate of rabbit antibodies to mice IgG with horseradish peroxidase. When staining, 3.3′-Diaminobenzidine was used as substrate.

### 2.5. Ethics Statement

All experimental procedures in mice were made to minimize animal suffering and carried out in line with the principles of humanity described in the relevant Guidelines of the European Community and Helsinki Declaration. The protocols were approved by the Institutional Animal Care and Use Committee (IACUC) affiliated with State Research Center of Virology and Biotechnology “Vector” (Permit Number: SRC VB “Vector”/10-05.2016). 

### 2.6. Immunization of Experimental Animals Ethics Statement

When immunizing, we used 5–6-week-old BALB/c mice (female) of weight 16–18 g from the State Research Center of Virology and Biotechnology Vector vivarium. Animals were divided into four groups with 5–10 mice in each group including (1) pE-CTL+pE-Th—mice immunized with a mix of DNA-vaccine pEV.CTL and pEV.Th encoding CTL- and Th-epitopes of Ebola virus, respectively; (2) pE-CTL—mice immunized with DNA plasmid pEV.CTL encoding CTL-epitopes of Ebola virus; (3) pDNA3.1—mice immunized with vector plasmid pDNA3.1 (negative control); and (4)—intact non-immunized animals to whom phosphate buffered saline was inoculated (PBS) (pH 7.6) (negative control). Mice were immunized three times intramuscularly with 100 μg DNA vaccine pEV.CTL or pEV.CTL + pEV.Th at 2-week intervals. An equivalent dose of pcDNA3.1 vector plasmid was used for mice from the control group. Two weeks after the last immunization, spleens were removed in animals and splenocytes were isolated to analyze T-cell immune response.

### 2.7. Detection of T-Cell Immune Response Using IFNγ ELISpot and Intracellular Cytokine Staining (ICS) Assay

Enzyme-Linked ImmunoSpot (ELISpot) and intracellular cytokine staining (ICS) assays were used to characterize the immune response of mice after immunization with DNA vaccines. Stimulation of splenocytes was carried out using a mix of synthetic peptides (KFINKLDALH, NYNGLLSSI, PGPAKFSLL, YFTFDLTALK, EYLFEVDNL, LFLRATTEL, and LYDRLASTV) from the compound of the designed antigens. Peptides were synthesized by Synpeptide Co., Ltd. (Shanghai, China) with >80% purity. Analysis of IFNγ ELISpot was performed with Mouse IFN-γ ELISPOT Set (BD, cat 551083, San Diego, CA, USA) according to the manufacturer’s instruction and as previously described [29]. To stimulate splenocytes, we used a mix of peptides at concentration 20 μg/mL of each peptide to 1 × 10^6^ cells followed by co-cultivation for 24 h. IFNγ-producing cells were calculated using an ELISpot-analyzer (Zeiss, Germany). ICS was performed according to the standard protocol of BD Biosciences as previously described [30]. To stimulate splenocytes, we used a mix of peptides at concentration 20 μg/ml of each peptide to 1 × 10^6^ cells and incubated for 20 h at 37 °C and 5 % CO_2_ and additionally for 5 h with Brefeldin A. Cells were washed with PBS and permeabilized with Cytofix/Cytoperm™ Plus Fixation/Permeabilization Kit (BD Biosciences, San Diego, CA, USA). When staining, the following monoclonal antibodies were used: PerCP Rat Anti-Mouse CD4, FITC Rat Anti-Mouse CD8a, PE Hamster Anti-Mouse CD3ε, and APC Rat Anti-Mouse IFN-γ (BD Pharmingen, San Diego, CA, USA). The samples were analyzed using flow cytometer FACSCalibur (Becton Dickinson, San Jose, CA 95131, USA) and Cell Quest software. 

### 2.8. Statistical Analysis

Statistical analysis of the obtained results was carried out with the R software environment for statistical analysis (version 3.2; https://www.R-project.org/). To evaluate the significance of the differences among the groups, the Kruskal–Wallis test was applied. Pair-wise distribution comparison of the analyzed indices in the experimental and control groups was conducted using one-sided Mann–Whitney test. When multiple testing, FDR procedure was performed to correct *p*-values.

## 3. Results and Discussion

### 3.1. Strategies to Design Polyepitope T-Cell Antigens

To stimulate response of CD8+ T-lymphocytes, viral antigens must be presented to CTL precursors not as full-length molecules, but as short peptides (8–12 amino acid residues) in complex with MHC class I molecules. These epitopes are formed from endogenously synthesized viral antigens in the result of proteasome-mediated processing and then are transferred to ER lumina by means of a transporter associated with antigen processing (TAP) proteins where it binds to emerged MHC class I molecules (see for review [31,32]). Since proteasome-mediated processing functions for antigens synthesized intracellularly, a vaccine inducing T-cell response may be designed as DNA vaccine because in this case the CTL vaccine epitopes are presented in the most natural way—through MHC class I-dependent antigen presentation pathway [33].

Unlike stimulation of CTLs, while stimulating CD4+ T-lymphocytes-helpers response, antigen should be presented to those cells in complex with MHC class II molecules. Usually, antigen processing and presentation occurs for extracellular antigens which are delivered in cells via endocytosis and phagocytosis. In this case, antigen processing takes place in the lysosome.

Thus, when designing artificial polyepitope T-cell immunogens capable of inducing responses of CD4+ and CD8+ T-lymphocytes to all epitopes it comprises, it is necessary to provide efficient proteasome- and/or lysosome-mediated processing of the expression product of the target gene by MHC class I and II pathway.

Different strategies can help achieve this goal:

(1) To combine epitopes in the compound of poly-CTL-epitope construct one may use spacer sequences comprising sites of proteasomal cleavage [34,35,36] and/or motifs for binding to TAP [37,38].
(2) To combine epitopes in the compound of poly-Th-epitope construct one may use motif [KR][KR] which is a cleavage site for a number of lysosomal cathepsins involved in antigen processing [39,40].
(3) To direct polyepitope immunogen to proteasome and to present CTL-epitopes to CD8+ T-lymphocytes by MHC class I pathway, genetic binding of ubiquitin sequence to its N- or C-terminus is typically used [41].
(4) To degrade polyepitope immunogen and present released Th-epitopes to CD4+ T-lymphocytes by MHC class II pathway, genetic binding of sequence of LAMP-1 (Lysosomal-associated membrane protein 1) tyrosine motif to its C-terminus is typically used to direct polyepitope immunogen from the secretory pathway to the lysosome [42,43,44,45].

**Figure 1:**
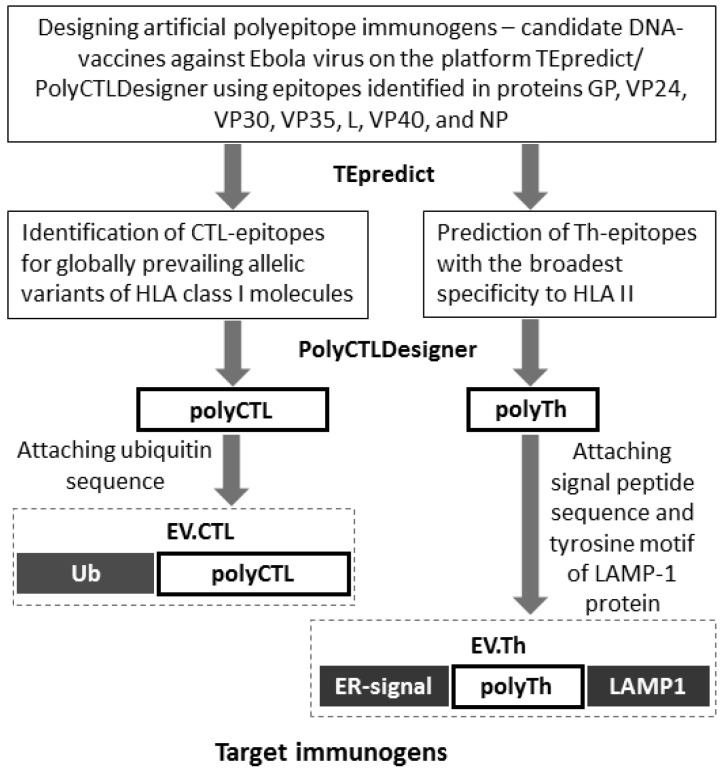
Designing artificial polyepitope antigens of Ebola virus.

In our study, two artificial polyepitope T-cell immunogens were designed, one of which comprises cytotoxic (CTL) and the other—T-helper (Th) epitopes identified in Ebola virus proteins GP, VP24, VP30, VP35, L, VP40, and NP (Figure 1). Previously we showed that adding ubiquitin to N-terminus of polyepitope antigen induces CD8+ T-cell response more efficiently as compared to adding the signal peptide and the LAMP-1 C-terminal fragment [30]. Therefore, we added N-terminal ubiquitin to the final poly-CTL-epitope construct, and poly-Th-epitope immunogen was designed using N-terminal signal peptide and LAMP-1 C-terminal fragment. N-terminal signal peptide should direct the polyepitope to the endoplasmic reticulum (ER), and C-terminal fragment of LAMP-1 should redirect the polyepitope from the secretory pathway to the lysosome.

### 3.2. Design of Artificial Poly-CTL-Epitope Antigen of Ebola Virus

For the purposes of designing poly-CTL-epitope antigen (EV.CTL), we used Immune Epitope Database (http://iedb.org) [24] to select known T-cell epitopes and peptide fragments of antigens of different Ebola virus strains with an experimentally verified capacity to bind to different allomorphs of MHC molecules. In total, at the time of antigen designing (2016) the database contained information on 1134 unique peptides from 65 antigens of 16 Ebola virus strains verified for their capacity to bind to 60 allomorphs of MHC class I molecules (56 Human Leukocyte Antigen (HLA) allelic variants). To analyze conservation of peptides, we used 14,556 amino acid sequences from NCBI ProteinBank (ncbi.nlm.nih.gov/protein) belonging to different Ebola viruses (Zaire ebolavirus, Sudan ebolavirus, Bundibugyo ebolavirus, Tai Forrest ebolavirus, and Reston ebolavirus). We considered peptides with experimentally verified cytotoxic activity. Furthermore, when designing target immunogens, we selected those with sufficiently high binding affinity to different HLA class I molecule variants (pIC50 > 6.3).

After that, we selected peptides identified at least in 1000 known viral sequences and interacting with at least two allelic HLA molecule variants. In total, we selected 44 peptides which cumulatively were restricted by 34 allelic HLA class I molecule variants including the most globally widespread ones (Table 1). It is known that optimally selected epitopes restricted by ten different HLA class I alleles cover virtually the entire population of any geographic region [46,47]. 

Based on the selected T-cell epitopes, we designed EV.CTL poly-CTL-epitope antigen using TEpredict/PolyCTLDesigner software we developed earlier [25,26] that we regard as a universal platform for rational design of polyepitope immunogens—candidate DNA vaccines to induce T-cell immunity to infectious as well as oncological diseases. PolyCTLDesigner enables us to select a minimal set of epitopes with known or predicted specificity to different allelic variants of MHC class I molecules covering a selected repertoire of HLA alleles with a preset degree of redundancy. After that, PolyCTLDesigner predicts binding affinity to TAP for the selected set of known or predicted epitopes using a model developed by Peters et al. [48] and when required adds TAP-specific amino acid residues (no more than three) to epitope N-terminus to optimize binding.

At the next step, PolyCTLDesigner analyzes all possible matchings of the selected peptides and detects the optimal spacer sequence for each pair providing an appropriate cleavage of epitopes with a release of proximal peptide C-terminus. To predict proteasomal and/or immunoproteasomal cleavage, PolyCTLDesigner uses models developed by Toes, et al. [49].

When analyzing matchings of epitopes, PolyCTLDesigner forms a direct graph where nodes denote epitopes and ribs correspond to acceptable matchings. Each rib has a relevant weight vector characterized by effective proteasomal cleavage, spacer length, and a number of predicted non-target epitopes at the joint. At the final stage, the software designs the optimal resultant of polyepitope immunogen sequence determined as a full simple way in the formed graph with the least length (weight).

In this study, we used PolyCTLDesigner to predict binding affinity of the selected peptides (Table 1) to TAP; when required software added alanine residue to peptides N-terminus to enhance interaction efficiency. Poly-CTL-epitope fragment of EV.CTL was designed using a degenerated spacer motif [ARSP][DLIT][LGA][VKA] with optimization of proteasomal cleavage and 10% exactness of proteasomal filter.

To test the immunogenicity of the designed vaccine construct in mice using ELISpot and ICS, we selected seven additional peptides with proven ability to induce cytotoxic response of T-lymphocytes in BALB/c mice: *KFINKLDALH, NYNGLLSSI, PGPAKFSLL, YFTFDLTALK, EYLFEVDNL, LFLRATTEL, and LYDRLASTV*. Based on the selected peptides, mouse polyepitope fragment included at C-terminus of the polyepitope construct was designed with PolyCTLDesigner. To verify synthesis of the designed antigen in transfected cells, we included C-terminal marker epitope EPFRDYVDRFYKTLR of p24 HIV-1 protein recognized by monoclonal antibodies 29F2 in the final construct.

Designed amino acid sequence appears as follows (mouse epitopes are italicized):

**MMVIFRLMR**—**ADLS**—GHMMVIFRL—**KK**—VQLPQYFTF—**ADLS**—KQIPIWLPL—**RK**—EYAPFARLL—RVPTVFHKK—FIYFGKKQY—**R**—VLYHRYNLV—**ADL**—YQGDYKLFL—AFPRCRYVHK—ATPVMSRFAA—AFAEGVVAFL—KVYWAGIEF—**R**—TVAPPAPVY—TLASIGTAF—**R**—TTIGEWAFW—**RK**—LANETTQAL—FLLQLNETI—**R**—FVHSGFIYF—**K**—IISDLSIFI—**R**—NFFHASLAY—**RR**—LANPTADDF—**K**—ILMNFHQKK—**ADLS**—FTPQFLLQL—YSGNIVHRY—**ADLA**—RTSFFLWVI—RTFSILNRK—**RK**—LSDLCNFLV—**ADLV**—HMMVIFRLM—**ADLK**—IMYDHLPGF—ALPQYFTFDL—YLEGHGFRF—**R**—FLSFASLFL—**R**—TRSFTTHFL—RLMRTNFLI—**ADG**—FRLMRTNFL—**R**—GQFLSFASL—**R**—SFASLFLPK—RLASTVIYR—ARLSSPIVL—AHPLARTAKV—QFLSFASLF—**R**—GYLEGTRTL—**R**—FRYEFTAPF—**KK**—*YFTFDLTALK—EYLFEVDNL—**R**—PGPAKFSLL—**RK**—LFLRATTEL—**RK**—NYNGLLSSI—**R**—LYDRLASTV—**R**—KFINKLDALH*—SGSG—**EPFRDYVDRFYKTLR**

The length of the designed polyepitope EV.CTL is 547 amino acids; a share of spacer sequences is 12.76%. To target polyepitope immunogen into proteasome, we added ubiquitin sequence to N-terminus of the final poly-CTL-epitope construct.

### 3.3. Design of Poly-Th-Epitope Ebola Virus

To achieve the most efficient induction of T-cell immune response, one should induce not only responses of CD8+ but also CD4+ T-lymphocytes; therefore, in the following steps, we constructed poly-Th-epitope fragment (EV.Th). We used Th-epitopes predicted for humans and showing the broadest specificity regarding HLA class II molecules. For the purpose, TEpredict [25] predicted Th-epitopes in Ebola virus proteins. PolyCTLDesigner [26] was used to select eight fragments of the length of 35–40 amino acid residues comprising the most of the Th-epitopes with the broadest specificity regarding different HLA class II allomorphs. N-terminus of the selected peptides was extended up to 5 amino acid residues as compared to the beginning of the first epitope, and C-terminus—up to 5 amino acid residues as compared to the end of the last epitope (Table 2).

Additionally included at C-terminus of the construct: universal Th-epitope PADRE (PAn DR Epitope)—AKFVAAWTLKAAA; marker epitope EPFRDYVDRFYKTLR of p24 HIV-1 protein recognized by monoclonal antibodies 29F2, and a C-terminal fragment of LAMP-1 protein—RKRSHAGYQTI. According to the literature, adding the signal peptide concurrently with LAMP-1 C-terminus fragment to the target antigen raises the level of CD4+ T-lymphocyte response significantly [50,51,52,53]. As a signal peptide, we selected the sequence of an N-terminal fragment of Ebola virus surface glycoprotein comprising MGVTGILQLPRDR leader peptide. Using the SignalP server [54] we predicted that the leader peptide in the designed artificial polypeptide is functional and should efficiently split out. Poly-Th-epitope antigen EV.Th was designed using K/R-K/R spacer sequences that form cleavage sites by lysosomal cathepsins [39,40]:

***MGVTGILQLPRDR***—FKRTSFFLWVIILFQRTFSIPLGVIHNSTLQVSDVDKL—**RR**—TNTNHFNMRTQRVKEQLSLKMLSLIRSNILKFINKLDA—**RR**—LTLDNFLYYLTTQIHNLPHRSLRILKPTFKHASVMSRL—**RR**—TQTYHFIRTAKGRITKLVNDYLKFFLIVQALKHNGTWQAE—**RR**—WDRQSLIMFITAFLNIALQLPCESSAVVVSGLRTLVPQSD—**RR**—SSAFILEAMVNVISGPKVLMKQIPIWLPLGVADQKTYSF—**RR**—QYPTAWQSVGHMMVIFRLMRTNFLIKFLLIHQGMHMVAGH—**RR**—ESADSFLLMLCLHHAYQGDYKLFLESGAVKYLE—**RR**—AKFVAAWTLKAAA—SGSG—**EPFRDYVDRFYKTLR—**SGSG—**RKRSHAGYQTI**

MGVTGILQLPRDR—signal peptide; AKFVAAWTLKAAA—PADRE epitope; EPFRDYVDRFYKTLR—marker epitope; RKRSHAGYQTI—C-terminal fragment of LAMP-1 protein.

### 3.4. Designing Artificial Genes and Producing Recombinant Plasmids—Candidate DNA Vaccines Against Ebola Virus Encoding Polyepitope Immunogens of Ebola Virus

Artificial genes encoding EV.CTL and EV.Th-immunogens of Ebola virus were designed using GeneDesigner software [27]. Reverse translation of amino acid sequences was conducted considering the frequency of codons in humans [55]. Kozak sequence (CCGCCACC) is located ahead of ATG initiating codon. At the end of the encoding sequence, three stop-codons (TAGTGATGA) were added. Designed genes—*EV.CTL* and *EV.Th* were synthesized and cloned in pcDNA 3.1 vector plasmid. As the result, we constructed two recombinant plasmids pEV.CTL and pEV.Th—candidate DNA vaccines against Ebola virus.

### 3.5. Analysis of Target Gene Expression

The genes expression of DNA vaccines was evaluated with two methods: specific mRNA synthesis assay and immunostaining of the transfected cells. To evaluate synthesis of specific mRNA, we isolated total RNA from 293T cells transfected with plasmids pEV.CTL and pEV.Th and obtained cDNA in RT. The obtained cDNA was used to carry out PCR using pairs of primers (f^CTL^, r^CTL^) and (f^Th^, r^Th^) to genes *EV.CTL* and *EV.Th*, respectively.

**Figure 2:**
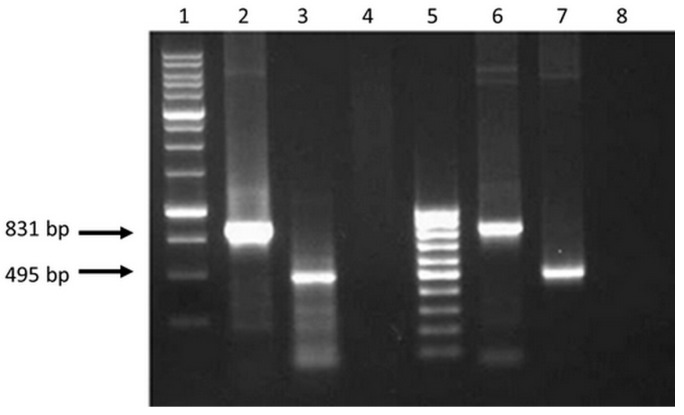
Electrophoregram on 1% agarose gel of PCR products: 1 - Molecular weight marker (M12, SibEnzyme); 2 and 3—PCR fragments of 831 and 495 bps obtained using cDNA as a matrix with primers (fCTL, rCTL) and (fTh, rTh), respectively; 4 and 8—The results of PCR with primers (fCTL, rCTL) and (fTh, rTh) and total RNA isolated from 293T cells transfected with plasmids pEV.CTL and pEV.Th, respectively (without reverse transcription; control for the absence of target plasmids in isolated samples of total RNA); 5—Molecular weight marker (M15, SibEnzyme); 6 and 7—PCR fragments obtained using plasmids pEV.CTL and pEV.Th as a matrix, respectively (positive control).

The results in Figure 2 demonstrate that the sizes of the amplified fragments correspond to the theoretically calculated sizes of amplification products, i.e., 891 bps for *EV.CTL* gene and 495 bps for *EV.Th* gene. Similar PCR fragments were obtained when using initial target plasmids pEV.CTL and pEV.Th (positive control) as a matrix. The findings indicate presence of specific mRNA in the total cell RNA fraction.

**Figure 3:**
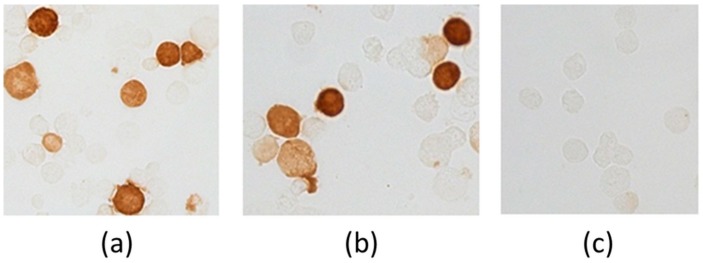
Evidence of genes expression in cells transfected with plasmids pEV.CTL and pEV.Th by immunohistochemical staining. (**a**) 293T-cells transfected with p*EV.CTL* plasmid. (**b**) 293T-cells transfected with p*EV.Th* plasmid. (**c**) 293T-cells transfected with pcDNA3.1 vector plasmid.

Immunohistochemical staining of cells transfected with pEV.CTL and pEV.Th plasmids was evaluated using MAT 29F2/30A6 antibodies to EPFRDYVDRFYKTL marker epitope, included in all constructs. The results depicted in Figure 3 demonstrate the presence of specific proteins. The findings confirm the expression of the target genes both at the level of transcription and translation.

### 3.6. Immunogenicity Study of DNA-Vaccine Constructs Encoding Multiple T-Cell Epitopes of Ebola Virus

Immunogenicity of the target DNA vaccine constructs was evaluated regarding their capacity to induce a T-cell response in BALB/c mice 14 days after the third immunization. The level of T-cell immune response was detected using IFNγ-ELISpot and ICS.

**Figure 4:**
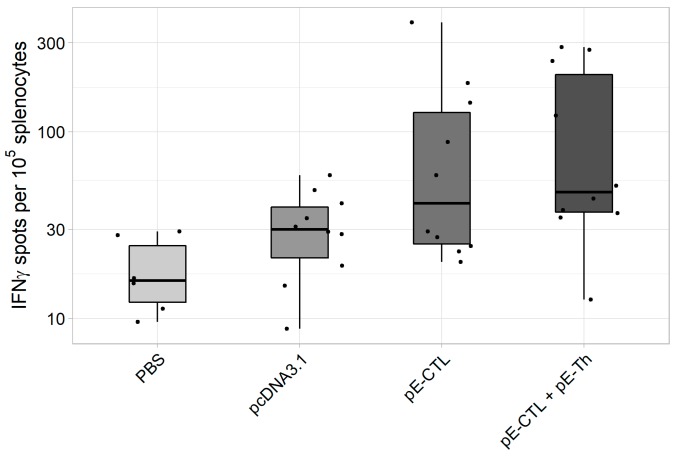
The results of IFNγ-producing T-cell count in IFNγ-ELISpot assay in BALB/c mice immunized with DNA-vaccine constructs encoding target immunogens (n = 6 for phosphate buffer solution (PBS) control group and n = 10 for the other groups). The figure represents spot count (i.e., IFNγ-producing T-cells) in different experimental and control animal groups.

ELISpot results (Figure 4) demonstrate that the induction of specific response was registered in both experimental groups [pE-CTL+pE-Th] and [pE-CTL], especially in the animal group immunized with a mix of target DNA vaccine constructs [pE-CTL+pE-Th]. Significant differences from both negative controls were observed only in [pE-CTL+pE-Th] group (Table 3).

**Figure 5:**
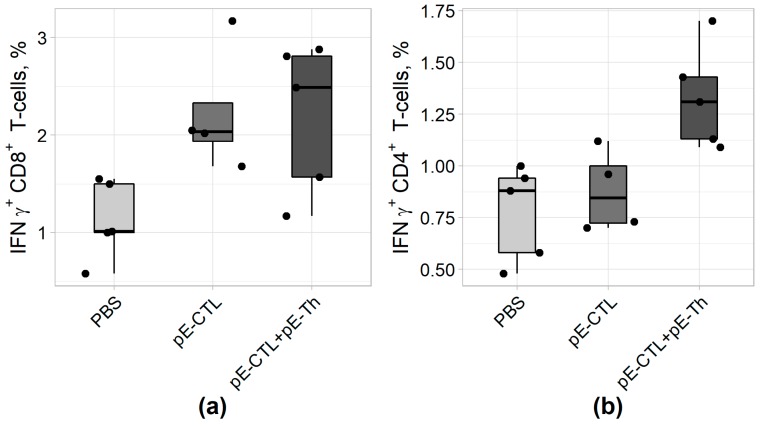
The results of IFNγ-producing CD4+ (A) and CD8+ (B) T-cell count using intracellular cytokine staining (ICS) approach in BALB/c mice immunized with DNA vaccine constructs encoding target immunogens (n = 5).

The capacity of vaccine constructs to induce IFNγ-producing CD4+ and CD8+ T-cells was tested by ICS after stimulating splenocytes with specific peptides. The results of ICS (Figure 5) revealed that statistically significant difference from control (Table 4) was demonstrated by IFNγ-producing CD8+ T-lymphocytes in animal groups immunized both with pEV.CTL and a mix (pEV.CTL + pEV.Th) DNA vaccines as well as by IFNγ-producing CD4+ T-helpers in the group immunized with only a mix of vaccine constructs (pEV.CTL + pEV.Th). The maximal responses of IFNγ-producing CD8+ T-lymphocytes (*p* = 0.024) and CD4 + T-cells (*p* = 0.012) were registered in the animal group immunized with a mix of vaccine constructs. This is believed to be caused by the synergistic effect of CD8+ and CD4+ T-lymphocytes.

To design the target antigens, we used PolyCTLDesigner software that we had developed for rational design of artificial polyepitope vaccine constructs [26]. It enables us to calculate amino acid sequence of polyepitope antigen by detecting the best spacer sequences for each pair of epitopes and optimal relative positions of epitopes in the construct considering state-of-the-art knowledge about the specificity of proteasomal processing of antigens and interaction between peptides and TAP. The findings revealed that the designed artificial DNA vaccine constructs encoding CTL and Th-epitopes of Ebola virus antigens provide expression of the target genes, as well as induce virus-specific responses of CD4+ and CD8+ T-lymphocytes in immunized mice.

## 4. Conclusions

Our original developed TEpredict/PolyCTLDesigner software was used in the study to predict cytotoxic and T-helper epitopes in a compound of seven Ebola virus proteins (GP, VP24, VP30, VP35, L, VP40, and NP) and to design two polyepitope immunogens EV.CTL and EV.Th on the base of those epitopes. Recombinant plasmids, candidate DNA vaccines against Ebola virus encoding the designed antigens, were obtained. We show that the designed DNA vaccine constructs provide a synthesis of corresponding mRNA and proteins in a eukaryotic cell culture, as well as induce statistically significant responses both of CD4+ and CD8+ T-lymphocytes in immunized animals, and consequently are promising candidates for further studies of their capacity to induce cytotoxic and protective responses. 

## Figures and Tables

**Table 1:** Predicted CD8+ cytotoxic T-lymphocytes (CTL)-epitopes in the sequences of Ebola virus proteins (antigens).

No.	Epitope	Antigen	Epitope Frequency	HLA Class I Alleles
1	ARLSSPIVL	L	1741	B*27:05; B*39:01; C*07:02
2	EYAPFARLL	NP	1764	A*24:03; A*24:02
3	FAEGVVAFL	GP	3881	B*39:01; A*02:01
4	FIYFGKKQY	L	1737	B*15:01; A*01:01; B*15:17
5	FLLQLNETI	GP	3862	A*02:01; A*24:02
6	FLSFASLFL	NP	1755	A*02:01; A*24:02; C*03:03
7	FPRCRYVHK	GP	3956	B*07:02; B*08:01
8	FRLMRTNFL	NP	1767	B*39:01; B*08:01; C*06:02
9	FRYEFTAPF	L	1744	B*39:01; C*14:02
10	FTPQFLLQL	GP	3868	A*02:01; A*24:02
11	FVHSGFIYF	L	1739	A*24:03;A*23:01;B*35:01;A*26:02;B*15:01; A*02:06; C*03:03
12	GHMMVIFRL	NP	1768	B*39:01; A*02:01; A*24:02
13	GQFLSFASL	NP	1753	B*15:01; B*27:05
14	GYLEGTRTL	L	1748	A*24:03; A*23:01
15	HMMVIFRLM	NP	1768	A*02:01; A*24:02
16	HPLARTAKV	NP	1766	B*07:02; B*51:01
17	IISDLSIFI	L	1713	A*02:01; A*69:01
18	ILMNFHQKK	NP	1711	A*03:01; A*11:01
19	IMYDHLPGF	VP35	1737	B*58:01; C*12:03
20	KQIPIWLPL	VP40	1766	B*40:01; B*27:05
21	KVYWAGIEF	VP24	1702	B*15:01; B*35:01; C*14:02
22	LANETTQAL	GP	1242	B*07:02; B*35:01; C*03:03
23	LANPTADDF	VP30	1686	B*35:01; B*58:01
24	LPQYFTFDL	VP40	1763	B*07:02; B*35:01
25	LSDLCNFLV	VP24	1725	A*01:01; C*05:01
26	MMVIFRLMR	NP	1768	A*03:01; A*11:01
27	NFFHASLAY	L	1750	B*15:01; B*35:01
28	QFLSFASLF	NP	1755	A*24:03; A*24:02
29	RLASTVIYR	GP	3947	A*03:01; A*31:01
30	RLMRTNFLI	NP	1766	A*02:01; A*24:02
31	RTFSILNRK	GP	1207	A*03:01; A*11:01; A*31:01
32	RTSFFLWVI	GP	3802	A*02:01; A*24:02
33	RVPTVFHKK	VP30	1684	A*03:01; A*31:01
34	SFASLFLPK	NP	1756	A*03:01; A*11:01
35	TLASIGTAF	L	1743	B*15:01; B*35:01
36	TPVMSRFAA	L	1738	B*07:02; B*35:01
37	TRSFTTHFL	L	1747	B*39:01; C*06:02
38	TTIGEWAFW	GP	3824	A*24:02; B*58:01; A*68:23; A*32:15; A*32:07
39	TVAPPAPVY	NP	1684	A*11:01; B*35:01
40	VLYHRYNLV	L	1746	A*02:01; A*03:19
41	VQLPQYFTF	VP40	1763	B*15:01; A*24:03
42	YLEGHGFRF	NP	1739	A*02:01; A*24:02
43	YQGDYKLFL	NP	1705	A*02:01; A*24:02
44	YSGNIVHRY	L	1750	A*01:01; B*58:01

**Table 2:** Predicted CD4+ T-helper (Th)-epitopes in the sequences of Ebola virus proteins (antigens)*.

Peptide	Protein	Fragment	Number of HLA-DR Allomorphs	Number of Epitopes
FKRTSFFLWVIILFQRTFSIPLGVIHNSTLQVSDVDKL	GP	14–51	48	11
TNTNHFNMRTQRVKEQLSLKMLSLIRSNILKFINKLDA	VP24	129–166	49	8
LTLDNFLYYLTTQIHNLPHRSLRILKPTFKHASVMSRL	L	1486–1523	50	5
TQTYHFIRTAKGRITKLVNDYLKFFLIVQALKHNGTWQAE	L	2111–2150	48	10
WDRQSLIMFITAFLNIALQLPCESSAVVVSGLRTLVPQSD	VP30	230–269	47	8
SSAFILEAMVNVISGPKVLMKQIPIWLPLGVADQKTYSF	VP40	70–108	42	8
QYPTAWQSVGHMMVIFRLMRTNFLIKFLLIHQGMHMVAGH	NP	186–225	50	13
ESADSFLLMLCLHHAYQGDYKLFLESGAVKYLE	NP	68–100	47	5

*—Table demonstrates peptide sequence, antigen name, the beginning and the end of the selected peptide, the number of HLA class II allomorphs interacting with a fragment, the number of Th epitopes predicted in a fragment.

**Table 3:** Results of statistical data analysis in ELISpot.

Animal Groups	Phosphate Buffer Solution (PBS)	pcDNA3.1	pE-CTL
pcDNA3.1	0.070	–	–
pE-CTL	0.029	0.148	–
pE-CTL + pE-Th	0.009	0.0296	0.264

**Table 4:** Statistical analysis results obtained using intracellular cytokine staining (ICS).

Animal Groups	CD8+IFNγ+		CD4+IFNγ+	
	PBS	pE-CTL	PBS	pE-CTL
pE-CTL	0.024	–	0.278	–
pE-CTL+pE-Th	0.024	0.635	0.012	0.024
